# EBNEO Commentary: Expectant Management Versus Medication for Patent Ductus Arteriosus in Preterm Infants—The PDA RCT

**DOI:** 10.1111/apa.70563

**Published:** 2026-05-02

**Authors:** Tim Hundscheid, Willem P. de Boode

**Affiliations:** ^1^ Radboud University Medical Center, Amalia Children's Hospital Nijmegen the Netherlands

## Abstract

Critically Appraised Summary Table (CAST) for the “Expectant Management vs Medication for Patent Ductus Arteriosus in Preterm Infants: The PDA Randomized Clinical Trial.”

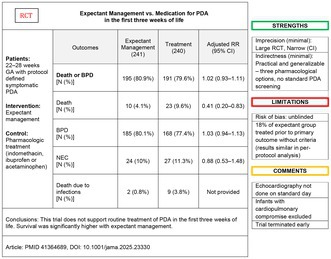

The trial by Laughon et al. echoes the findings of two recent trials [1, 2] in which relevant neonatal morbidities were also not positively influenced after pharmacological treatment for PDA. Apart from the lack of benefit, these findings add to the growing concerns on potential harm of pharmacological treatment with prostaglandin synthesis inhibitors, especially for an increased risk of death [3].

These previous trials focused on early, at 24 to 72 h postnatal age, pharmacological treatment specifically with ibuprofen. The current trial has both a longer time frame of enrollment and pharmacological treatment with the choice of treatment, dose and route of administration at the discretion of the clinical team. The administration of ibuprofen (35.4%), indomethacin (28.8%), acetaminophen (25.8%) or more than one of these medications (9.2%) in the active pharmacological treatment group reflects the clinical heterogeneity in neonatal care [4]. One major strength of this pragmatic approach is that the findings of the trial are potentially more generalizable. Nevertheless, since prophylactic indomethacin was used in 9 of 33 recruiting centers during the trial period, it would be interesting to subgroup centers that did and did not use prophylactic indomethacin.

Enrolling patients in PDA trials is challenging, as supported by the flowchart in Figure 1. Nevertheless, the trial succeeded in enrolling a substantial amount of the most immature infants, i.e., those with a gestational age below 26 weeks, often underrepresented in trials. Regarding hemodynamic significance of the PDA, this trial, in comparison to PDA diameter based inclusion in the BeNeDuctus and Baby‐OSCAR trial, [1, 2] used a protocol based definition of a symptomatic PDA [5]. Although helpful in better categorizing patients, the validity of this staging, in comparison to the PDA severity score, is questionable [6]. Whereas a PDA severity score based categorization might be the most sophisticated method to select the high‐risk babies for PDA treatment, i.e., those most likely to benefit from effective treatment, it requires echocardiographic expertise hampering the generalizability.

Another limitation of PDA trials is the inefficacy of pharmacological closure in a rather large proportion of infants. PDA closure rates are not reported in this study. Although clinical outcomes are far more relevant than achieving PDA closure per se, this information would be interesting. Moreover, since findings from the pilot PDA‐RCT, albeit limited in sample size, suggest that those with successful closure of their PDA have a lower risk for death or BPD at 36 weeks' PMA [7].

The PDA RCT by Laughon et al. further supports the current guideline from the American Association of Paediatrics to refrain from routine treatment of a PDA within the first 2 weeks of life [8]. Alongside the growing evidence from numerous RCTs on PDA management, there is also growing adherence to an interventional closure strategy, which is more effective than pharmacological treatment in closing the PDA and causes less morbidity than surgical ligation [9]. However, prior to the widespread use of this therapeutic intervention, a trial that proves overall benefit of this successful closure strategy as compared to a truly expectant management is of the utmost importance.

URL LINK: https://ebneo.org/ebneo‐commentary‐expectant‐management‐versus‐medication‐for‐patent‐ductus‐arteriosus‐in‐preterm‐infants‐the‐pda‐rct/.

## Author Contributions

**Tim Hundscheid:** conceptualization, writing – original draft, writing – review and editing. **Willem P. de Boode:** writing – review and editing, supervision.

## Funding

TH received a personal Clinical fellowship 2024 grant from the Netherlands Organization for Health Research (ZonMw; project number 09032242410047).

## Conflicts of Interest

The authors declare no conflicts of interest.

## Data Availability

Data sharing not applicable to this article as no datasets were generated or analysed during the current study.
